# Polytobacco usage and mental health among Malaysian secondary school-going adolescents: Findings from the national school-based study

**DOI:** 10.18332/tid/204789

**Published:** 2025-07-04

**Authors:** Kuang Hock Lim, Yoon Ling Cheong, Jia Hui Lim, Chee Cheong Kee, Sumarni Mohd Ghazali, Yong Kang Cheah, Pei Pei Heng, Ali Aman Marine, Mohd Hazilas Mat Hashim, Wei Wen Goh, Hui Li Lim

**Affiliations:** 1 Institute for Medical Research, National Institutes of Health, Ministry of Health Malaysia, Shah Alam, Malaysia; 2 School of Pharmacy, Faculty of Health and Medical Sciences, Taylor’s University, Subang Jaya, Malaysia; 3 Biostatistics and Data Repository Sector, National Institutes of Health, Ministry of Health Malaysia, Shah Alam, Malaysia; 4 School of Economics, Finance and Banking, University Utara Malaysia, Sintok, Malaysia; 5 Institute for Public Health, National Institutes of Health, Ministry of Health Malaysia, Shah Alam, Malaysia; 6 Faculty of Medicine and Health Science, University Putra Malaysia, Serdang, Malaysia; 7 Institute for Clinical Research, National Institutes of Health, Ministry of Health Malaysia, Shah Alam, Malaysia

**Keywords:** smoking, adolescents, tobacco use, mental health, polytobacco consumption

## Abstract

**INTRODUCTION:**

The emergence of novel tobacco products has led to an increase in the consumption of diverse tobacco items among adolescents. The smoking habits of adolescents adversely affect their physical and emotional health. This study aimed to ascertain the prevalence of polytobacco use among Malaysian teenagers and to identify the mental health issues associated with dual or polytobacco use.

**METHODS:**

This is a secondary dataset analysis of cross-sectional data from 27479 secondary school adolescents who participated in the 2017 Malaysian Adolescent School Health survey. The primary outcome of our study pertained to self-reported mental health characteristics (stress and depressive symptoms). The research used multivariable logistic regression analysis to assess the associations between the use of tobacco products and mental wellness.

**RESULTS:**

Of the respondents, 79.9% were non-tobacco users, 9.2% were single tobacco users, 4.7% were dual tobacco users, and 7.3% were polytobacco users. Individuals engaged in polytobacco use exhibited elevated levels of stress (16.7%; p<0.001) and depression (33.7%; p<0.001) relative to those utilizing fewer tobacco products. Polytobacco product users exhibited a 1.64-fold (95% CI: 1.30–2.06) increased likelihood of reporting stress symptoms and a 1.75-fold (95% CI: 1.46–2.09) increased likelihood of reporting depression symptoms in comparison to non-tobacco users.

**CONCLUSIONS:**

Adolescents who engage in dual or polytobacco use frequently experience internalized mental health issues, including symptoms of stress and despair. It is essential to perform early assessments of high-risk individuals, provide them with information regarding the importance of cessation, and implement proactive interventions for these groups' psychological challenges.

## INTRODUCTION

The introduction of novel tobacco products, such as electronic cigarettes (ECs), has significantly transformed the global landscape of teenage tobacco consumption over the past decade. This shift has led to a notable rise in dual (use of two types of tobacco products) or polytobacco product use (use of three or more tobacco products), with ECs playing a central role in this trend^1,2^. The use of multiple tobacco products – referred to as poly use – is increasingly recognized as a significant public health concern^3,4^. Compared to conventional cigarettes, alternative tobacco products like e-cigarettes may pose a lower risk of exposing others to harmful substances. However, these products are often used in social settings, driven by appealing flavors, peer influence, and prevailing trends^5^.

Many adolescents are drawn to various tobacco products under the misconception that they are less harmful or can assist with smoking cessation^6^. For instance, a 2020 study found that 5.4% of Minnesota students had used multiple tobacco products in the past 30 days, most commonly combining ECs and conventional cigarettes (28.3%), followed by ECs and cigars (19.9%) and a mix of ECs, cigars, and cigarettes (10.2%)^7^. Similarly, the 2019 Korea Youth Risk Behavior Web-based Survey (KYRBWS) revealed that 53.1% of Korean adolescent smokers used a single tobacco product, while 24.8% used two, and 22.1% used more than two. Between 2016 and 2018, although conventional cigarette use among adolescent males remained steady, polytobacco use increased from 2.94% to 3.32%, and the proportion of smokers using multiple products rose from 30.4% to 35.4%^8^.

In Malaysia, Lim et al.^9^ found that 6.5% of school-going adolescents were dual or polytobacco users. Of these, 53.7% used both cigarettes and e-cigarettes, while 23.7% used cigarettes, e-cigarettes, and shisha^9^. The prevalence of dual/poly use was notably higher among males, older adolescents, Malays, natives of Sabah and Sarawak, those with lower perceived harm of tobacco use, individuals lacking tobacco education in school, and those exposed to secondhand smoke at home, outdoors, or in school.

The growing prevalence of dual tobacco use among adolescents is concerning, as it is associated with a heightened risk of nicotine dependence compared to single-product use. Young people are especially vulnerable to nicotine addiction, and those engaging in dual or poly use often display more intense dependence symptoms – such as cravings and urges – than exclusive users of conventional cigarettes^10,11^. Additionally, dual/poly users are more likely to engage in other risky behaviors, including the use of alcohol, cannabis, and other illicit substances^12^.

Tobacco use is also strongly linked to mental health challenges, particularly neuropsychiatric conditions such as depression^13^. Smoking can exacerbate depressive symptoms through stress, chronic health conditions, and the biological impact of nicotine^13^. Research shows that smoking is associated with increased anxiety and the onset of depressive symptoms in both adults^14,15^ and adolescents^16^. This finding is especially troubling during adolescence – a critical developmental stage – since behaviors formed during this period often persist into adulthood. Notably, around 75% of adolescents experiencing significant stress and depressive symptoms are likely to face depression later in life^17^.

Given this context, there is an urgent need for research on the effects of dual/poly tobacco use in Malaysia. Such research would equip policymakers with crucial evidence to formulate targeted youth smoking prevention strategies and provide healthcare professionals with local data to support health promotion efforts. Furthermore, it would enhance the global understanding of tobacco-related issues by contributing insights from a developing country facing health challenges for decades.

This study aims to examine dual and polytobacco use among adolescents in Malaysia and its association with mental health outcomes, particularly stress and depression. Based on existing literature, we hypothesize that both single and dual/polytobacco use have significant impacts on adolescent mental health. The study seeks to generate meaningful data on adolescent behavior and well-being by exploring these associations, potentially shaping future public health policies and interventions.

## METHODS

### Sample and study design

This is a secondary dataset analyses of the data from the National Health and Morbidity Survey 2017: Adolescent Health Survey (NHMS 2017: AHS). This study employed a cross-sectional and multistage sampling technique to get a representative sample of adolescents aged 13–17 years attending secondary schools in Malaysia. The initial phase entailed the stratification of Malaysian states, which was succeeded by additional stratification within each state according to urban and rural classifications. The Ministry of Education and Rural and Regional Development supplied the sampling frame utilizing 2016 school enrolment data. The primary sampling unit consisted of 212 secondary schools in Malaysia, chosen using systematic random sampling^18^. The secondary sampling unit was a selection of 4 to 10 classes from each chosen school using proportionate size sampling. All students from the selected class were invited to participate in the study. The sample size was calculated using a variance of interest of 3%, a margin of error of 3%, and a 95% confidence interval, using a design effect of 2 from the 2012 GSHS-Malaysia study. A non-response rate of 25% was projected, necessitating a sample size of 30496 respondents. Only 27497 were included in the analysis. Yielding a 90.1% response rate. The remaining 2999 (9.9%) were not included due to respondents refusing to participate in the study, being absent during the interview, or providing incomplete answers.

Active consent was used for data collection from the chosen respondents. A letter outlining the study’s aims, responder confidentiality, and the consent form was dispatched to parents/guardians via the school administration. Participation was permitted solely for respondents who submitted the completed consent form. Data were gathered in certain school zones without the involvement of school personnel before the survey session. The research assistants, who were instructed on the study’s aims, collected the data. Participation was optional, and participants were notified that their data would be utilized exclusively for research purposes. The study team assisted respondents with clarification on particular items in the questionnaire. The study protocol received permission from the Ministry of Health and the Ministry of Education in Malaysia, and ethical clearance was obtained from the Medical Research and Ethical Committee (NMRR-16-698-30042).

### Instrument

The questionnaire was adopted from the Malaysia Global School Health Survey 2012, which had been validated in prior research. The dependent variables, depression and stress symptoms were evaluated using the validated Malay version of the DASS 21 questionnaire. Seven items were used to measure anxiety and depression symptoms, respectively. The instrument has shown high reliability among Malaysian youth (Cronbach’s alpha of 0.90 for stress and 0.917 for measuring depression symptoms), respectively^19^. Respondents were asked to rate how frequently they experienced certain feelings over the past week, e.g. stress (I tended to overreact to situations) and depression (I found it difficult to get motivated to do things), with response options ranging from ‘never’ (0) to ‘almost always’ (3). The scores were then multiplied by 2. Respondents who scored between 0 and 13 were classified as not experiencing stress symptoms, whilst those who scored 10 or higher were categorized as having depressive symptoms during the last seven days^20^.

### Variables

The independent variables are current tobacco status, which was measured by asking, ‘During the last 30 days: 1) Did you smoke cigarettes?; 2) Did you use traditional hand-rolled cigarettes?; 3) Did you use roll-your-own cigarette paper?; 4) Did you use a cigar?; 5) Did you use pipe smoking?; 6) Did you use an e-cigarette or vape?; 7) Did you use chewing tobacco?; and 8) Did you use snuff?’. The respondents who responded ‘no’ to all of the items were classified as ‘tobacco non-users’ those who answered ‘yes’ to any single item were categorized as ‘mono tobacco users’, and the respondents who answered ‘yes’ to any two or more items were classified as ‘dual/poly users of a tobacco product’.

### Covariates

Gender, ethnicity (Malay, Chinese, Indian, Bumiputra Sabah, Bumiputra Sarawak, and others), age group (13–15 years, 16–17 years), parental/guardian marital status (married, divorced), history of physical attack at least once during last 12 months (yes, no), involvement in physical fights at least once during previous 12 months (yes, no), the experience of physical abuse at least once during last 12 months (yes, no), the experience of verbal abuse at least once during previous 12 months (yes, no), experience of bullying at least once during last 12 months (yes, no), exposure to secondhand smoke (SHS) in the past week (yes, no), parental supervision most of the time (yes, no), and have close friend(s) (yes, no).

### Statistical analysis

The data underwent two rounds of cleaning to ensure quality: first by a research team member in the field, and then by the research management team at the institutional level. We randomly selected 10% of the data and compared it with the hard copy records to verify consistency. Frequency analysis was performed on each variable to identify missing or outlier values. The data were weighted according to the selection probabilities, considering the inverse probability of selecting each school, the inverse of selecting each classroom, and non-response adjustment factors at both the school and student levels, calculated by classroom. Descriptive statistics were used to summarize the sociodemographic characteristics of the participants. A chi-squared analysis examined the relationship between exposure and the dependent variable (exposure to SHS)^16^. The multivariable logistic regression models were fitted with all independent variables with a p≤0.25 in bivariate analyses. The main effect’s adjusted odds ratio (AOR) and 95% confidence interval (CI) were calculated. The model was also assessed for potential two-way (multiplicative) interactions between the independent variable (no, mono, and dual/poly tobacco use, with all the covariates). All statistical analyses were performed using the SPSS statistics program version 2018, with the complex samples module, applying a two-tailed test at a 95% significance level.

## RESULTS

Males comprised about 50% of the sample, and over 60% of those surveyed were aged 13–15 years, and Malay. Nearly 20% of respondents were current tobacco users, more than half of the respondents were schooled in urban areas, 87.4% of respondents were from intact families, >95% of respondents had close friends, and only 13% of respondents reported supervision by the parents/guardian most of the time (Table 1).

**Table 1:** Sociodemographic characteristics of school-going adolescents who participated in the National Health and Morbidity Survey 2017: Adolescent Health Survey (NHMS 2017: AHS) (N=27497)

*Characteristics*	*Estimated population*	*Sample*	*%*	*95% CI*
**Gender**				
Male	1064953	13135	49.6	46.5–52.7
Female	1081492	14362	50.4	47.3–53.5
**Locality**				
Urban	1210741	15899	56.4	52.8–60.0
Rural	935705	11598	46.6	40.0–47.2
**Ethnicity**				
Malay	1354538	18713	63.1	57.7–68.2
Chinese	358504	4100	16.7	13.4–20.6
Indian	149227	1428	7.0	5.2–9.3
Bumiputra Sabah	149354	1781	7.0	4.3–11.1
Bumiputra Sarawak	96823	924	4.5	2.6–7.6
Other	37997	554	1.8	1.3–2.5
**Age** (years)				
13–15	1302499	16952	60.7	56.8–64.5
16–17	843946	10545	39.3	35.6–43.2
**Marital status of parents**				
Married	1834475	23544	87.4	86.7–88.0
Divorce/separated	264521	3387	12.6	12.0–13.3
**Physical attack at least once**				
Yes	542467	6735	25.3	24.1–26.6
No	1600544	20722	74.7	73.4–75.9
**Physical abuse at least once**				
Yes	253449	3034	11.8	10.8–12.9
No	1888965	24416	88.2	87.1–89.2
**Physical fight at least once**				
Yes	533138	6584	24.9	23.6–26.2
No	1609941	20877	75.1	72.8–76.4
**Bullied at least once**				
Yes	346882	4436	16.2	15.2–17.2
No	1795518	23022	83.8	82.8–84.8
**Verbal abuse at least once**				
Yes	924222	11681	43.2	41.8–44.7
No	1214101	15727	56.8	55.3–58.2
**Having a close friend**				
Yes	2058223	25424	96.4	96.8–95.9
No	77103	942	3.6	3.2–4.1
**Current tobacco use**				
No	1674730	21699	78.9	77.7–80.0
Mono	194349	2458	9.2	8.6–9.7
Dual/poly	254742	3066	12.0	11.1–12.9
**Exposed to SHS**				
Yes	900560	11385	42.0	40.5–43.6
No	1242418	16070	58.0	56.4–59.5
**Supervision by parents most of the time**				
Yes	282898	3713	13.2	12.5–13.9
No	1860778	23473	86.8	86.1–87.5

The sample was weighted based on the probability of selecting the school, classroom response rate of school level, class, and student, and post-stratification adjustment on gender and form of the students.

The study found that 19.8% (95 % CI: 19.0–20.7) of respondents reported stress symptoms during the last seven days. A significantly higher proportion (1.52-fold) of stress symptoms exposure was observed among current dual/poly tobacco users (28.1%; 95% CI: 25.8–30.5 vs 18.4%; 95% CI: 17.5–19.4) than tobacco non-users. Female respondents of Indian and Chinese descent reported higher stress symptoms compared to their counterparts (male and Malay). In addition, respondents who were schooling in rural areas reported lower stress symptoms in the last week than those schooling in urban areas. Furthermore, those who had been bullied once, physically abused, or physically attacked at least once, reported higher significant levels of stress symptoms compared to those who had never been bullied/physically abused/physically attacked (Table 2).

**Table 2:** Association of tobacco use (no, mono, dual/poly) and stress symptoms among secondary school adolescents in Malaysia among participants in the National Health and Morbidity Survey 2017: Adolescent Health Survey (NHMS 2017: AHS) (N=27497)

*Variables*	*Stress symptoms*								
	*Yes*				*No*				
	*Estimated population*	*Sample*	*%*	*95% CI*	*Estimated population*	*Sample*	*%*	*95% CI*	*p*
**Gender**									
Male	194089	2331	18.8	17.7–19.9	838700	10443	81.2	80.1–82.3	0.003
Female	219976	2921	20.9	19.8–22.0	833211	11103	79.1	78.2–80.2	
**Locality**									
Urban	247451	3218	20.9	19.7–22.2	937639	12366	79.1	77.8–80.3	0.006
Rural	166614	2034	18.5	17.4–19.6	734272	9180	81.5	80.4–82.6	
**Ethnicity**									
Malay	242325	3402	18.4	17.4–19.4	1074653	14856	81.6	80.6–82.6	<0.001
Chinese	71821	795	20.4	18.5–22.5	279875	3226	79.6	77.5–81.5	
Indian	37635	341	26.1	22.6–29.9	106747	1045	73.9	70.1–77.4	
Bumiputra Sabah	34841	414	24.4	21.3–27.5	108023	1297	75.6	72.5–78.5	
Bumiputra Sarawak	19211	183	20.4	17.1–24.1	75041	716	79.6	75.9–82.9	
Other	8230	117	23.0	18.6–28.1	27570	406	77.0	71.9–81.4	
**Age** (years)									
13–15	240436	3121	19.2	17.9–20.1	1026004	13407	81.0	79.9–82.1	0.016
16–17	173629	2131	21.2	19.9–22.6	645907	8139	78.8	77.4–80.1	
**Marital status of parents**									
Married	347353	4392	19.5	18.6–20.4	1436656	18575	80.5	79.6–81.4	0.006
Divorce/separated	56207	725	22.0	20.2–23.8	199745	2557	78.0	76.2–79.8	
**Physical attack at least once**									
Yes	147306	1816	28.0	25.4–29.7	378823	4734	72.0	70.3–73.5	<0.001
No	266194	3428	17.1	16.2–18.0	1290732	16784	82.9	82.0–83.8	
**Physical abuse at least once**									
Yes	82086	1007	33.6	31.3–36.1	162072	1931	66.4	63.9–68.7	<0.001
No	330556	4231	18.0	17.1–18.9	1507644	19587	82.0	81.1–82.4	
**Physical fight at least once**									
Yes	143550	1764	27.8	26.2–29.4	372590	4630	72.8	70.6–73.8	<0.001
No	269685	3480	17.2	16.4–18.1	1297299	16892	82.8	81.9–83.6	
**Bullied at least once**									
Yes	113802	1459	34.1	32.0–36.2	220279	2843	65.9	63.8–68.0	<0.001
No	299597	3786	17.1	16.3–18.0	1448935	18676	82.9	82.0–83.7	
**Verbal abuse at least once**									
Yes	241255	3112	26.9	25.6–28.2	657208	8270	73.1	71.8–74.4	<0.001
No	171063	2121	14.5	13.7–15.4	1008503	13211	85.5	84.6–86.3	
**Having a close friend**									
Yes	391134	4941	19.5	18.7–20.4	1610249	20829	80.5	79.6–81.3	
No	20916	283	28.2	24.5–32.1	53378	626	71.8	67.9–75.5	
**Parental supervision most of the time**									
Yes	54228	637	19.8	17.9–21.9	219178	2968	80.2	78.1–82.1	0.993
No	359292	4608	19.8	19.0–20.7	1451294	18555	80.2	79.3–81.0	
**Current tobacco use**									
No	301089	3890	18.4	17.5–19.4	1333040	17328	81.6	80.6–82.5	<0.001
Mono	40167	519	21.5	19.3–23.9	146602	1858	78.5	76.1–80.7	
Dual/poly	68521	788	28.1	25.8–30.5	175537	2156	70.9	69.5–74.2	
**Exposed to SHS**									
Yes	197752	2447	22.6	21.4–23.9	675927	8632	77.4	76.1–78.6	<0.001
No	171063	2798	17.8	16.9–18.8	993888	12886	82.2	81.2–83.1	

The sample was weighted based on the probability of selecting the school, classroom response rate of school level, class, and student, and post-stratification adjustment on gender and form of the students. Chi-squared analysis in complex sampling was used to determine the association between stress symptom and independent variables.

Table 3 shows that nearly one-third of the respondents reported having depression symptoms during the last week (32.8%; 95% CI: 31.7–33.9). Almost half (46.8%) of the dual/poly tobacco users reported depression symptoms compared to mono-users and non-users of tobacco products (p<0.001). The other covariates, except the age group, also showed significant levels of depression symptoms.

**Table 3:** Association of tobacco use (no, mono, dual/poly) and depressive symptoms among secondary school adolescents in Malaysia among participants in the National Health and Morbidity Survey 2017: Adolescent Health Survey (NHMS 2017: AHS) (N=27497)

*Variables*	*Depression symptoms*								
	*Yes*				*No*				
	*Estimated population*	*Sample*	*%*	*95% CI*	*Estimated population*	*Sample*	*%*	*95% CI*	*p*
**Gender**									
Male	333015	4010	32.2	30.6–33.8	701615	8783	67.8	66.2–69.4	0.233
Female	352643	4615	33.4	32.0–34.8	703833	9437	66.6	65.2–68.0	
**Ethnicity**									
Malay	395731	5547	30.0	28.7–31.3	923856	12731	70.0	68.7–71.3	<0.001
Chinese	124375	1378	35.3	32.8–38.6	227530	2647	64.7	62.0–67.2	
Indian	67888	597	47.2	42.2–52.2	75944	787	52.8	47.8–57.8	
Bumiputra Sabah	56203	637	38.8	35.0–42.8	88548	1089	61.2	57.2–65.0	
Bumiputra Sarawak	28286	269	29.9	26.3–33.8	66317	633	70.1	66.2–73.7	
Other	13180	197	36.2	29.7–43.2	23251	333	63.8	56.8–70.3	
**Age** (years)									
13–15	412135	5317	32.5	31.1–34.0	855645	11237	67.5	66.0–68.9	0.557
16–17	273524	3308	33.2	31.4–35.1	549803	6983	66.8	64.9–68.6	
**Marital status of parents**									
Married	564903	7142	31.6	30.5–32.7	1223229	15871	68.4	67.3–69.5	<0.001
Divorce/separated	99149	1222	38.6	36.2–41.1	157445	2057	61.4	58.9–63.8	
**Physical attack at least once**									
Yes	234551	2856	44.5	42.5–46.6	292105	3700	55.5	53.4–57.5	<0.001
No	449947	5752	28.8	27.7–29.9	1111662	14502	71.2	70.1–72.3	
**Physical fight at least once**									
Yes	234055	2821	45.4	43.3–47.5	281967	3579	54.6	52.5–56.7	<0.001
No	450384	5789	28.8	27.6–29.7	1121790	14624	71.4	70.3–72.4	
**Physical abuse at least once**									
Yes	133484	1594	54.3	51.6–57.0	112153	1347	45.7	43.0–48.0	<0.001
No	550250	7009	29.9	28.8–31.0	1291568	16852	70.1	69.0–71.2	
**Bullied at least once**									
Yes	176318	2213	52.7	50.2–55.2	158514	2088	47.3	44.8–49.8	<0.001
No	506808	6391	28.9	27.9–30.0	1245696	16117	71.1	70.0–72.1	
**Verbal abuse at least once**									
Yes	380703	4788	42.3	40.7–43.9	519523	6612	57.7	56.1–59.3	<0.001
No	301959	3802	25.5	24.3–26.8	881282	11561	74.5	73.2–75.7	
**Having a close friend**									
Yes	38399	479	51.3	46.6–56.5	36120	433	48.5	43.5–53.4	<0.001
No	643301	8095	32.1	31.0–33.2	1363026	17718	67.9	66.8–69.0	
**Parental supervision most of the time**									
Yes	258986	3316	28.0	26.7–29.3	666098	8946	72.0	70.7–73.3	0.015
No	723733	5299	36.6	35.1–38.1	737632	9252	63.4	61.9–64.9	
**Current tobacco use**									
No	494108	6349	30.2	29.1–31.3	1141676	14884	69.8	68.7–70.9	<0.001
Mono	67105	854	35.6	32.8–38.4	121469	1539	64.4	61.6–67.2	
Dual/poly	115014	1311	46.8	43.4–50.2	130709	1651	53.2	49.8–56.6	
**Exposed to SHS**									
Yes	315753	3889	36.0	34.4–37.6	561332	7218	64.0	62.4–65.6	<0.001
No	368566	4721	30.4	29.2–31.7	842456	10981	69.6	68.3–70.8	

The sample was weighted based on the probability of selecting the school, classroom response rate of school level, class, and student, and post-stratification adjustment on gender and form of the students. Rao-Scott chi-squared test in complex sampling was used to determine the association between depression symptom and independent variables. The study was cross-sectional.

Multivariable analysis was used to obtain the odds of dual/poly users of tobacco products (Table 4). The study showed that the odds of stress (AOR=1.37; 95% CI: 1.20–1.57) and depression (AOR=1.59; 95% CI: 1.38–1.83) symptoms were significantly higher among polytobacco users. There is no significant difference between stress symptoms between mono-users and non-users of tobacco products. However, dual users of tobacco products showed higher levels of stress and depression symptoms compared to mono-user and non-tobacco users.

**Table 4:** Multivariable logistic regression analysis of tobacco use (no, mono, dual/poly) among school-going and mental well-being (stress symptoms, depression symptoms) among adolescents’ participants in the National Health and Morbidity Survey 2022: Adolescent Health Survey (NHMS 2017: AHS) (N=27497)

*Variables*	*Stress symptoms*		*Depression symptoms*	
	*AOR*	*95% CI*	*AOR*	*95% CI*
**Gender**				
Male ®	1		1	
Female	1.33	1.21–1.45	1.24	1.13–1.35
**Locality**				
Urban	1.19	1.07–1.33	1.06	0.96–1.17
Rural ®	1		1	
**Ethnicity**				
Malay ®	1		1	
Chinese	1.23	1.05–1.44	1.45	1.27–1.66
Indian	1.25	1.02–1.54	1.81	1.52–2.17
Bumiputra Sabah	1.32	1.13–1.55	1.33	1.13–1.57
Bumiputra Sarawak	1.06	0.85–1.35	0.85	0.70–1.04
Other	1.20	0.90–1.59	1.23	0.91–1.66
**Age** (years)				
13–15 ®	1			
16–17	1.27	1.14–1.41		
**Marital status of parents**				
Married ®	1		1	
Divorce/separated	1.03	0.92–1.15	1.24	1.11–1.37
**Physical attack at least once**				
Yes	1.30	1.15–1.44	1.22	1.10–1.36
No ®	1		1	
**Physical fight at least once**				
Yes	1.22	1.10–1.35	1.25	1.14–1.37
No ®	1		1	
**Physical abuse at least once**				
Yes	1.25	1.10–1.42	1.40	1.24–1.59
No ®	1		1	
**Verbal abuse at least once**				
Yes	1.70	1.56–1.86	1.67	1.53–1.81
No ®	1		1	
**Bullied at least once**				
Yes	1.80	1.60–2.01	1.81	1.61–2.03
No ®	1		1	
**Having close friend**				
Yes ®	1		1	
No	1.29	1.04–1.60	2.00	1.31–3.04
**Current tobacco use**				
No ®	1		1	
Mono	1.10	0.94–1.29	1.18	1.03–1.36
Dual/poly	1.37	1.20–1.57	1.59	1.38–1.83
**Exposed to SHS**				
Yes	1.18	1.07–1.30	1.14	1.06–1.23
No ®	1		1	
**Supervision by parents most of the time**				
Yes ®			1	
No			1.18	1.06–1.32

AOR: adjusted odds ratio. ® Reference categories.

**Figure 1:**
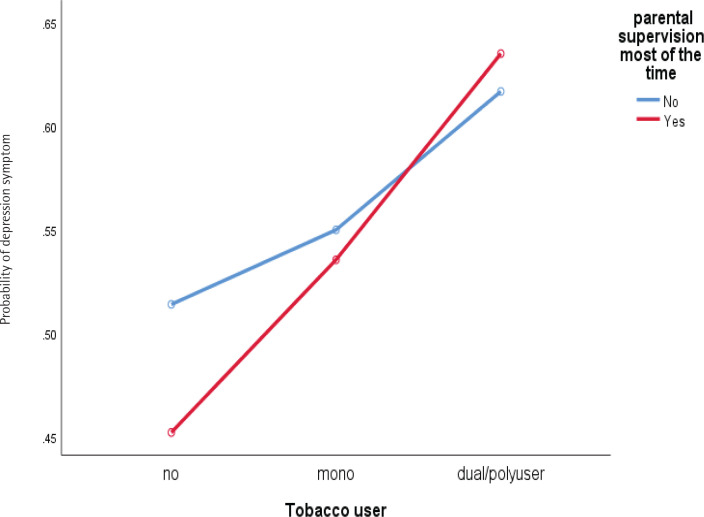
Interaction between tobacco users and parental supervision most of the time with depression symptom among school-going adolescents who participated in the National Health and Morbidity Survey: Adolescent Health Survey (NHMS: AHS) 2017

**Figure 2:**
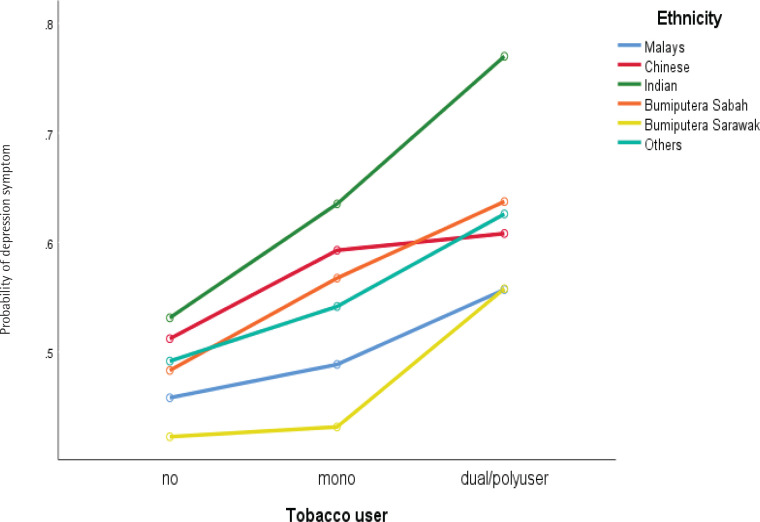
Interaction between tobacco users and ethnic groups with depression symptoms among school-going adolescents who participated in the National Health and Morbidity Survey: Adolescent Health Survey (NHMS: AHS) 2017

**Figure 3:**
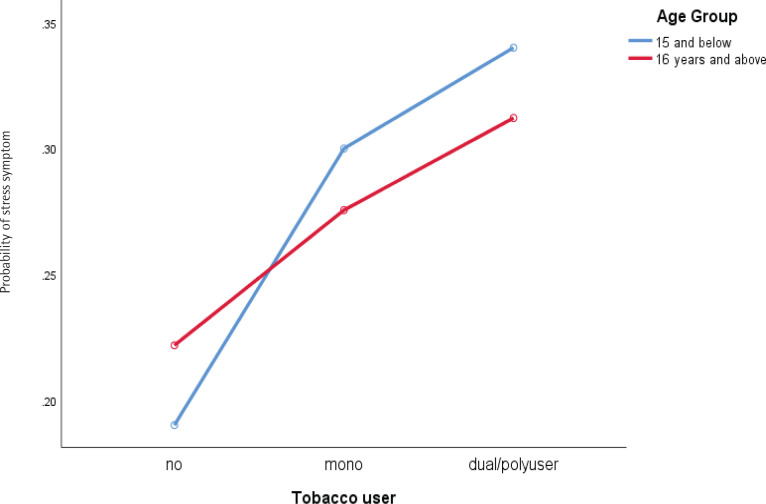
Interaction between tobacco users and age groups with stress symptoms among school-going adolescents who participated in the National Health and Morbidity Survey: Adolescent Health Survey (NHMS: AHS) 2017

**Figure 4:**
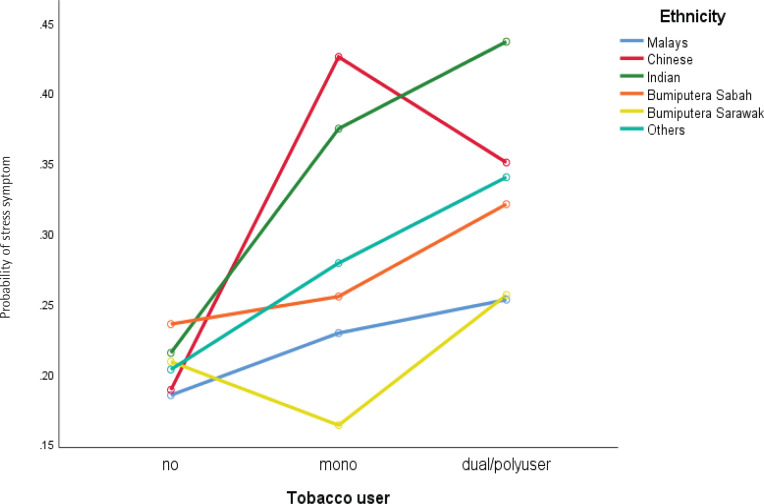
Interaction between tobacco users and ethnic groups with stress symptoms among school-going adolescents who participated in the National Health and Morbidity Survey: Adolescent Health Survey (NHMS: AHS) 2017

Significant two-way interaction was found for stress symptoms between ethnicity and dual/poly use (Figure 1), and the age group of respondents and dual/poly use of tobacco product (Figure 2), whilst ethnicity and dual/poly user supervision most of the time by parents/guardians and dual/poly user for depression symptoms, Figures 3 and 4 (see also Supplementary file).

## DISCUSSION

This study investigated the association between dual/poly tobacco use and stress and depression symptoms among secondary school adolescents in Malaysia. The findings suggest that individuals who use one, two, or multiple types of tobacco are more prone to mental health issues than non-smokers, with polytobacco use being primarily linked to a greater frequency of mental health concerns. Dual/poly users of tobacco products showed higher significant stress and depression symptoms during the last seven days compared to non-users of tobacco. This finding is consistent with the finding of Buheit et al.^15^ who reported that the use of tobacco products increased stress levels by 69% among the general population in Denmark. This finding might because nicotine dependence is higher among dual/poly tobacco users than among single tobacco users and non-users^21^. Given that nicotine dependence is linked to psychological distress^22^, the findings of this study may be explained by the deprivation reversal model, whereby nicotine abstinence is stressful, and nicotine exposure seems to increase stress levels and exacerbate mood disturbances by inducing changes in neural pathways and neurotransmitter systems that are implicated in mood regulation^23,24^.

The proportion of individuals experiencing depressive symptoms was significantly higher among dual or polytobacco users (46.8%; 95% CI: 43.4–50.2) compared to those who used only one type of tobacco product (35.6%; 95% CI: 32.8–38.4), and non-users (30.2%; 95% CI: 29.1–31.3). A clear dose-response relationship was observed, indicating that the number of tobacco products used was positively associated with both the prevalence and odds of depressive symptoms. These findings are consistent with Kwon et al.^16^ who reported a similar trend among Korean youth, where depression symptoms increased from 24.3% in non-users to 49.9% in polytobacco users. The adjusted odds ratios for depression also increased with the number of products used, from 1.58 for single-product users to 1.81 for dual users and 2.18 for polytobacco users.

This pattern aligns with earlier research by Pasco et al.^24^, which found an increased risk of major depressive disorder among smokers (age-adjusted OR=1.46; 95% CI: 1.03–2.07), with the odds more than doubling for heavy smokers (>20 cigarettes/day). Similarly, Needham^25^, in a longitudinal study of 10828 US adolescents, reported that higher initial cigarette use was associated with more significant increases in depressive symptoms over time. Both male and female adolescents with higher levels of initial substance use consistently reported elevated depressive symptoms across all measured time points. The systematic review of Fluharty et al.^14^ identified 51 studies linking tobacco use to depression symptoms, further supporting our findings. Kwon et al.^16^ further emphasized that polytobacco users were particularly susceptible to increased depressive symptoms. Additionally, a recent review of substance use and mental health among adolescents in the US and Canada found that smokers were 1.65 times more likely to experience depression and 2.21 times more likely to suffer from anxiety compared to non-smokers^26^. Consistent findings were also reported by Guo and Yan^27^ based on data from the National Health and Nutrition Examination Survey (2005–2018) in the United Kingdom, where smokers had the highest likelihood of experiencing depression (OR=1.94; p<0.01). A significant positive correlation was also found between daily smoking and depression (OR=1.66, p for trend <0.01)^27^. These results are further supported by the Population Assessment of Tobacco and Health (PATH) Study, which analyzed data from 13617 US youth aged 12–17 years and found that tobacco users were significantly more likely to report internalizing problems, including depressive symptoms (AOR=1.6; 95% CI: 1.4–1.8)^28^. As the number of tobacco products used increased, the severity of these mental health issues also worsened. The higher likelihood of depression among mono-users and polytobacco users may be explained by both biological and environmental factors. First, neurobiological research shows that nicotine exposure affects the dopamine system, both acutely and chronically, which can lead to long-term abnormalities in dopamine transport and lower dopamine levels, increasing the risk of depression^29^. Second, frequent tobacco use can be indicative of a stressful home or work environment. Chronic stress has been shown to exacerbate depression by impairing neuroplasticity and producing abnormal levels of neurotrophic factors^30^. Structural brain changes, such as loss of dendritic spines and synapses and reduced dendritic branching, have been linked to long-term stress and depression^31^. Smoking is also associated with various long-term medical disorders, such as asthma, which may predispose adolescents to develop depressive symptoms^32^.

This study found a significant interaction between age groups and tobacco users, where adolescents aged 13–15 years showed lower stress levels compared to their counterparts aged 16–17 years. Still, younger adolescents who used mono and dual/poly tobacco products showed higher stress levels. The finding of higher stress levels is in line with the literature, which states that adolescents aged ≥15 years face more new challenges in early adolescence^33^. Still, the use of tobacco products changes this situation. We postulate that this may be because adolescents aged 13–15 years are more addicted to nicotine since adolescents who start smoking at a younger age tend to be more addicted to tobacco products^34^; apart from that, this may also be due to their difficulty in obtaining tobacco products like their counterparts aged 16–17 years^35^, which may contribute to the stress levels faced by them given the level of addiction they face.

Our study also found that adolescents whose are always supervised by parents/guardians and who were dual/polytobacco users showed higher levels of depression. This finding may be due to respondents who were dual/polytobacco users knowing that their parents disapprove of their behavior and regular supervision makes them feel more anxious and afraid that their parents/guardians will understand their behavior causes anxiety and contributes to the depression symptoms experienced. However, our postulation should be investigated and refined in future studies^36^ (Wang et al.^16^). In addition, a significant interaction was found to occur on the dependent variable (stress and depression symptoms) due to respondents of Chinese descent showing high levels of stress and depression symptoms when using single tobacco products. Still, the levels of stress and depression symptoms decreased among adolescents who use dual/polytobacco products. In contrast, respondents from other ethnicities who use dual/polytobacco products showed increased stress and depression symptoms. These findings require an in-depth culturally qualitative study among adolescents who are single or dual/polytobacco users to be conducted in future studies. In all cases, reverse causality cannot be ruled out.

### Limitations

This study has several limitations. We used secondary data obtained through a cross-sectional design and self-reporting, which may lead to inaccuracies, as students could conceal their smoking behaviors and fail to report them accurately. As the data on tobacco use were self-reported, this may cause recall or response bias. Additionally, it is unclear whether polytobacco use is associated with all mental health-related variables, and cross-sectional studies are limited in their ability to establish causal relationships. The analysis is also limited by the inability to fully control residual confounding factors (such as underlying mental health problems). In addition, this study lacks biochemical validation to confirm smoking status, which means there may have been under-reporting of tobacco use.

Additionally, since the data were collected in 2017, they may not accurately reflect the current trends in dual/polytobacco use among youth in Malaysia^16^. Furthermore, the residual confounding was not controlled for and, lastly, the findings only apply to Malaysian school-going adolescents, and generalization cannot be applied to youth in other countries. However, a prior study in Malaysia found consistency between self-reported tobacco use and exhaled carbon monoxide levels when respondents’ anonymity and confidentiality were assured. Despite these limitations, the study highlights the association between increased dual/ polytobacco use and mental health issues among adolescents in Malaysia, where school-based health education on adolescent smoking is currently being implemented^16^.

## CONCLUSIONS

Dual and polytobacco use was linked to mental health risks during adolescence. Dual and polytobacco users are exposed to higher levels of nicotine, which may lead to the use of other tobacco products, creating a cycle of increased nicotine consumption and further mental health risks. This behavior may also serve as a gateway to more serious drug use^37^. Understanding the broader implications of polytobacco use in adolescents is essential for addressing these issues.

## Supplementary Material



## Data Availability

The data supporting this research are available from the authors on reasonable request.
