# Supplementary material for: Polytobacco usage and mental health among Malaysian secondary school-going adolescents: Findings from the national school-based study
Source: Tob Induc Dis. 2025 Jul 4;23:10.18332/tid/204789. doi: 10.18332/tid/204789 (PMC12228094; doi:10.18332/tid/204789)
Supplement: Supplementary file 1 [file TID-23-91-s1.pdf]

Supplementary File

INTERACTION BETWEEN INDEPENDENT VARIABLES FOR DEPRESSION SYMPTOMS		
**Variable**	**Variable**	**Percentage (95% CI)**
**Tobacco User**	**Ethnic**	
Non tobacco user	Malay	27.9(26.6-29.2)
Mono tobacco user	Malay	32.5(29.7-35.4)
Dual/poly tobacco user	Malay	41.4(37.7-44.7)
Non tobacco user	Chinese	33.5(30.9-36.3)
Mono tobacco user	Chinese	48.9(35.3-62.7)
Dual/poly tobacco user	Chinese	51.2(43.3-59.0)
Non tobacco user	Indian	40.3(36.0-44.8)
Mono tobacco user	Indian	57.7(42.7-71.4)
Dual/poly tobacco user	Indian	78.8(67.4-86.9)
Non tobacco user	Bumiputra Sabah	34.2(30.4-38.3)
Mono tobacco user	Bumiputra Sabah	43.2(34.2-52.7)
Dual/poly tobacco user	Bumiputra Sabah	53.1(44.5-61.6)
Non tobacco user	Bumiputra Sarawak	26.0(21.6-30.9)
Mono tobacco user	Bumiputra Sarawak	27.3(19.3-37.1)
Dual/poly tobacco user	Bumiputra Sarawak	42.0(32.6-52.0)
Non tobacco user	Others	32.4(26.1-39.4)
Mono tobacco user	Others	38.8(23.7-56.4)
Dual/poly tobacco user	Others	52.6(31.4-68.3)
**Tobacco User**	**Parental supervision most of the time**	
Non tobacco user	Yes	25.2(23.0-27.6)
Mono tobacco user	Yes	37.8(31.1-45.1)
Dual/poly tobacco user	Yes	53.8(47.3-50.2)
Non tobacco user	No	30.9(29.9-32.1)
Mono tobacco user	No	35.1(32.2-38.1)
Dual/poly tobacco user	No	45.7(42.2-49.2)
**INTERACTION BETWEEN INDEPENDENT VARIABLES FOR STRSS SYMPTOMS**		
**Tobacco User**	**Ethnic**	
Non tobacco user	Malay	17.4(16.3-18.6)
Mono tobacco user	Malay	19.3(17.0-22.0)
Dual/poly tobacco user	Malay	24.2(21.7-26.9)
Non tobacco user	Chinese	18.8(16.8-20.8)
Mono tobacco user	Chinese	39.0(28.0-51.2)
Dual/poly tobacco user	Chinese	35.6(29.2-42.5)
Non tobacco user	Indian	22.1(18.8-25.8)
Mono tobacco user	Indian	35.7(24.3-48.9)
Dual/poly tobacco user	Indian	43.4(34.4-52.9)

Non tobacco user	Bumiputra Sabah	22.8(19.4-26.5)
Mono tobacco user	Bumiputra Sabah	23.7(17.8-30.8)
Dual/poly tobacco user	Bumiputra Sabah	32.4(25.9-39.8)
Non tobacco user	Bumiputra Sarawak	19.9(15.7-24.9)
Mono tobacco user	Bumiputra Sarawak	12.5(6.6-22.4)
Dual/poly tobacco user	Bumiputra Sarawak	24.9(17.9-33.7)
Non tobacco user	Others	18.7(13.9-24.6)
Mono tobacco user	Others	26.8(16.5-40.4)
Dual/poly tobacco user	Others	41.9(27.3-58.1)
**Tobacco User**	**Age Group**	
Non tobacco user	13-15	17.1(16.0-18.3)
Mono tobacco user	13-15	22.4(199-25.1)
Dual/poly tobacco user	13-15	29.3(26.1-32.8)
Non tobacco user	16-17	20.5(19.0-22.1)
Mono tobacco user	16-17	20.2(16.5-24.6)
Dual/poly tobacco user	16-17	26.4(23.1-30.0)

© 2025 Lim K.H. et al.
